# Comparison Epidemiology between Tuberculosis and COVID-19 in East Java Province, Indonesia: An Analysis of Regional Surveillance Data in 2020

**DOI:** 10.3390/tropicalmed7060083

**Published:** 2022-05-27

**Authors:** Budi Utomo, Chow Khuen Chan, Ni Made Mertaniasih, Soedarsono Soedarsono, Shifa Fauziyah, Teguh Hari Sucipto, Febriana Aquaresta, Dwinka Syafira Eljatin, I Made Dwi Mertha Adnyana

**Affiliations:** 1 Department of Public Health and Preventive Medicine, Faculty of Medicine, Universitas Airlangga, Tambaksari, Surabaya 60132, East Java, Indonesia; 2 Department of Biomedical Engineering, Faculty of Engineering, Universiti Malaya, Kuala Lumpur 50803, Malaysia; ckchan@um.edu.my; 3 Department of Medical Microbiology, Faculty of Medicine, Universitas Airlangga, Tambaksari, Surabaya 60132, East Java, Indonesia; nmademertaniasih@gmail.com; 4 Tuberculosis Laboratory, Institute of Tropical Disease, Universitas Airlangga, Mulyorejo, Surabaya 60115, East Java, Indonesia; 5 Department of Pulmonology and Respiratory Medicine, Faculty of Medicine, Universitas Airlangga, Tambaksari, Surabaya 60132, East Java, Indonesia; ssoedarsono@gmail.com; 6 Akademi Analis Kesehatan Delima Husada Gresik, Gresik 61111, East Java, Indonesia; shifafauziyah1996@gmail.com; 7 Institute of Tropical Disease, Universitas Airlangga, Mulyorejo, Surabaya 60115, East Java, Indonesia; teguhharisucipto@staf.unair.ac.id; 8 Palembang Health Laboratory, Inspektur Yazid Street, Kemuning, Palembang 30126, South Sumatera, Indonesia; febriana.aquaresta@gmail.com; 9 Master Program in Tropical Medicine, Faculty of Medicine, Universitas Airlangga, Tambaksari, Surabaya 60132, East Java, Indonesia; deljatin92@yahoo.co.id (D.S.E.); dwikmertha13@gmail.com (I.M.D.M.A.)

**Keywords:** tuberculosis, COVID-19, infectious disease

## Abstract

Tuberculosis and COVID-19 are among the infectious diseases that constitute a public health concern. Therefore, this study aims to examine the recent epidemiology of tuberculosis and COVID-19 in East Java Province, Indonesia, in 2020. Case-based surveillance data were acquired with a retrospective design between January and December 2020 by the East Java Health Officer. The data were analyzed using Quantum Geographic Information System (QGIS) for mapping, and Microsoft Excel for recording. Furthermore, the statistical analysis (Spearman correlation test) was carried out via Statistical Package for Social Science (SPSS) applications. A total number of 38,089 confirmed cases of tuberculosis was recorded, with an incidence rate of 95.49/100,000 population, a case fatality rate (CFR) of 3.6%, and an average treatment success rate of 87.78%. COVID-19 is a new viral disease, with a total of 84,133 confirmed COVID-19 cases in East Java, with an incidence rate of 232.9/100,000 population. The highest incidence rate was found in Mojokerto city, while the lowest was found in Sampang. Furthermore, the CFR values of tuberculosis and COVID-19 were 1.4% and 6.8%, respectively. The regional survey in East Java Province showed that the incidence of tuberculosis remains high. This indicated that the search for active cases and preventive promotion was not completed. Therefore, inter-sectoral collaboration can be adapted to provide suitable tuberculosis health care.

## 1. Introduction

Coronavirus disease 2019 (COVID-19) was identified as an infectious illness caused by Coronavirus-2 Severe Acute Respiratory Syndrome (SARS-CoV-2). The daily increase in cases significantly affected the world, with 3.8 million fatalities reported in 2020; therefore, it was declared a pandemic by the World Health Organization [1].

During the pandemic threat, respiratory disorders were aggravated by COVID-19 infection. Due to the significant morbidity and mortality caused by COVID-19, several sectors in society attempted to avoid and conquer the pandemic. Pulmonary tuberculosis is caused by mycobacterium tuberculosis, which is transferred to people in close contact with patients, such as relatives, coworkers, colleagues, and friends, through coughing, talking, and sneezing [2]. This was particularly true for patients with pulmonary tuberculosis (TB), who are very susceptible to viral infection [3,4].

Studies on the existence of COVID-19 co-infection in pulmonary tuberculosis patients are important, due to the high risk and severity of COVID-19 disease. According to Coronel et al., tuberculosis patients have a very high risk of severe illness and mortality from COVID-19 [3]. Furthermore, incorrect treatment and hazardous conduct also increase the possibility of developing new diseases [4,5]. Therefore, this study aims to determine the epidemiology of the number of cases of pulmonary tuberculosis and COVID-19 in Indonesia, specifically in the province of East Java.

## 2. Materials and Methods

### 2.1. Study Design

The East Java Province is the eastern part of Java Island with a land area of 47,799.75 km^2^. It is located in 111°0′ to 114°4′ East Longitude (BT) and 7°12′ to 8°48′ South Latitude (LS) with four regional boundaries, namely, the north side (Java Sea), south, (Indian Ocean), west, (Central Java Province), and east (Bali Strait).

This was a retrospective cohort study using routinely regional surveillance data. The data were obtained from the Health Province Open Data Website, which offers free access to current epidemiological resources for infectious and non-communicable diseases (https://bit.ly/profil-kesehatan-2020 (accessed on 22 March 2021), including COVID-19 and TB [6]. The province consisted of 38 regencies, with a total population of 39,886,288, in accordance with the the East Java Health Profile. Therefore, the service was managed by East Java Health Officers from January to December 2020. Subsequently, the collected data were represented using graphs and geographical mapping for easier comprehension. COVID-19 cases were defined as all patients with or without symptoms of COVID-19 that were confirmed as positive using Reverse Transcriptase Polymerase Chain Reaction (RT-PCR) methods. Geographical mapping for the distribution of tuberculosis and COVID-19 cases was analyzed using Quantum Geographic Information System (QGIS) version 3.16.14-Hannover.

### 2.2. Statistical Analysis

All demographic data, together with tuberculosis and COVID-19 cases, were collected and analyzed using Statistical Package for Social Science software. The correlation between the incidence rate and the case fatality rate of tuberculosis and COVID-19 was calculated. An evaluation of the correlation between the case recovery rate and case fatality rate of tuberculosis, as well as the incidence rate and treatment success rate of tuberculosis, was also carried out. Furthermore, the correlation between treatment success rate and case fatality rate of tuberculosis, case recovery rate, and case fatality rate of COVID-19, was determined. We also calculated geographical factors, including the ratio of public health centres, number of doctors, nurses, midwifery, public health workers, environmental health workers, nutritionists, and pharmacists (Tables S1–S3). All the ratios were calculated per 100,000 of the population. These ratios were statistically tested by Pearson Correlation Test. For the sex distribution, the difference between both female and male patients with COVID-19 and tuberculosis was calculated. The data were analyzed using the Spearman correlation test, with a significance value of *p* < 0.05. 

### 2.3. Ethical Clearance

This study used routine/annual surveillance data from regional open platforms, which were provided by the Health Officer of East Java Province. The data were already anonymized; therefore, no ethical clearance was needed.

## 3. Results

### 3.1. The Incidence Rate (IR) of Tuberculosis and COVID-19

In this study, the incidence rate of tuberculosis in East Java Province in 2020 was 95.49/100,000 population (Table 1), with a varying case recovery rate (Table 2). The number of cases was higher among males than females (Table 3 and Table 4). The number for women in East Java in 2020 was 20,374,104, while the number for men was 20,291,592. Among all the residents in East Java, Probolinggo residents have a higher number of females (604) with tuberculosis than males (561). The highest infection rate was discovered in Madiun (296.51/100,000 population), while the lowest was proposed in Malang (0.07/100,000 population) (Table 1). When the data were analyzed according to sex, the incidence rate of tuberculosis in males (117.66/100,000 inhabitants) was higher than in females (93.49/100,000 inhabitants) (Table 3).

The regency with the highest tuberculosis incidence rate in males was Madiun (342.62/100,000 population), while the lowest rate was found in Pacitan (57.88/100,000 population) (Table 3). For females, the regency with the highest incidence rate of tuberculosis was Pasuruan (223.16/100,000 inhabitants) and the lowest rate was discovered in Pacitan (93.49/100,000 inhabitants) (Table 3). The highest incidence rate of COVID-19 infection was discovered in Mojokerto (789.9/100,000 populations), while the lowest was found in Sampang (52.5/100,000 populations) (Table 1). An analysis based on sex showed that the incidence rate of COVID-19 in females (215.74/100,000 inhabitants) was higher than in males (198.04/100,000 inhabitants). Meanwhile, the regency with the highest rate of males was Mojokerto (744.46/100,000 inhabitants), while the lowest rate was found in Sampang (48.58/100,000 inhabitants) (Table 3). The regency with the highest incidence rate of COVID-19 in females was Mojokerto (804.51/100,000 inhabitants), while the lowest rate was found in Madiun (51.14/100,000 inhabitants) (Table 4). 

### 3.2. The Case Fatality Rate (CFR) of Tuberculosis and COVID-19

The overall case fatality rate of tuberculosis in East Java Province in Indonesia was 3.6%. The highest CFR of TB infection was discovered in Probolinggo (7%), while the lowest was found in Surabaya (0.4%). Meanwhile, the highest CFR of COVID-19 infection was obtained in Pasuruan (11%), while the lowest was found in Tulungagung 2.1% (Table 1).

### 3.3. Case Recovery Rate (CRR) of Tuberculosis and COVID-19

The case recovery rate of tuberculosis was defined as patients with positive examination results upon treatment, bacteriological examination results at the end of treatment, and at one of the previous examinations.

The highest case recovery rate for tuberculosis was discovered in Magetan (96.6%), while the lowest was obtained in Batu City (11.3%). Meanwhile, the case recovery rate of COVID-19 was defined as COVID-19 patients with positive results at the beginning of RT-PCR, and negative results at the end of the examination. The highest case recovery rate of COVID-19 was found in Sidoarjo (92.6%), while the lowest was discovered in Tuban (64.5%) (Table 2).

### 3.4. The Treatment Success Rate (TSR) of Tuberculosis

The treatment success rate of tuberculosis is defined as the number of patients in all cured cases and complete treatment among treated and reported cases. In this study, the highest treatment success rate of tuberculosis was found in Magetan (95.97%), while the lowest was found in Bondowoso (65.89%) (Table 5).

### 3.5. The Age Group of COVID-19 Cases

**Figure 1:**
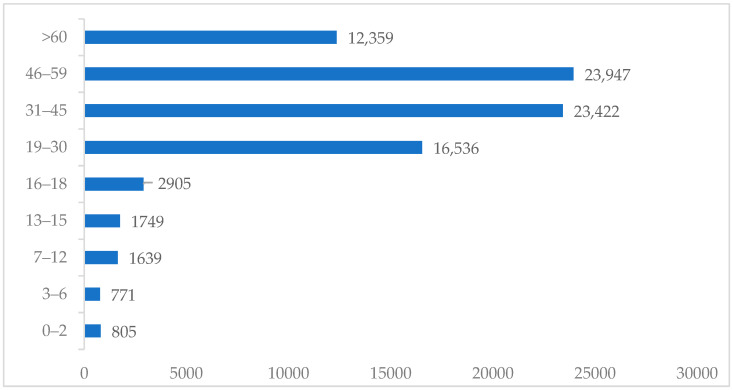
The distribution of COVID-19 cases based on age group.

Based on age group, the highest number of COVID-19 cases was found in those between 46 to 59 years old (23,947 individuals), while the lowest was found in those between 3 and 6 years old (771 individuals) (Figure 1).

### 3.6. The Correlation Test

The analysis of data with Spearman correlation showed no significant correlation between the incidence rate and case fatality rate of tuberculosis (*p* = 0.912, *p* > 0.05) (Table 6). It was also discovered that there was no significant correlation between incidence rate and case fatality rate for COVID-19 (*p* = 0.219, *p* > 0.05), the case recovery rate and case fatality rate for tuberculosis (*p* = 0.698, *p* > 0.05), the incidence rate and treatment success rate for tuberculosis (*p* = 0.795, *p* > 0.05), the treatment success rate and case fatality rate for tuberculosis (*p* = 0.659, *p* > 0.05), and the case recovery rate and case fatality rate for COVID-19 (*p* = 0.164, *p* > 0.05). The difference between the number of male and female patients with tuberculosis (*p* = 0.202, *p* > 0.05) and COVID-19 (*p* = 0.942, *p* > 0.05) was not significant (Table 6). In this study, we also calculated the geographical data consisting of the ratio number of public health centers, doctors, nurses, midwifery, public health workers, environmental health workers, nutritionists and pharmacists, and also the incidence rate of HIV in these areas (Tables S1–S3). Table 7 shows that the total number of health workers per 100,000 populations was significantly correlated with the incidence rate of tuberculosis (*p* < 0.05). These variables demonstrate a positive correlation with the tuberculosis incidence rate. Surprisingly, the incidence rate of HIV was also correlated with the incidence rate of TB (*p* < 0.05) (Table 7). However, the case recovery rate between TB and case recovery rate of COVID-19 was also significantly different (Table 8). While in COVID-19 aspect, the incidence rate of COVID-19 was also significantly correlated with the incidence rate of HIV and the number of health workers (doctor, nurse, public health workers, environmental health workers, nutritionist, and pharmacist) (Table 9). 

### 3.7. Interpretation by Quantum Geographic Information System (QGIS) Application

**Figure 2:**
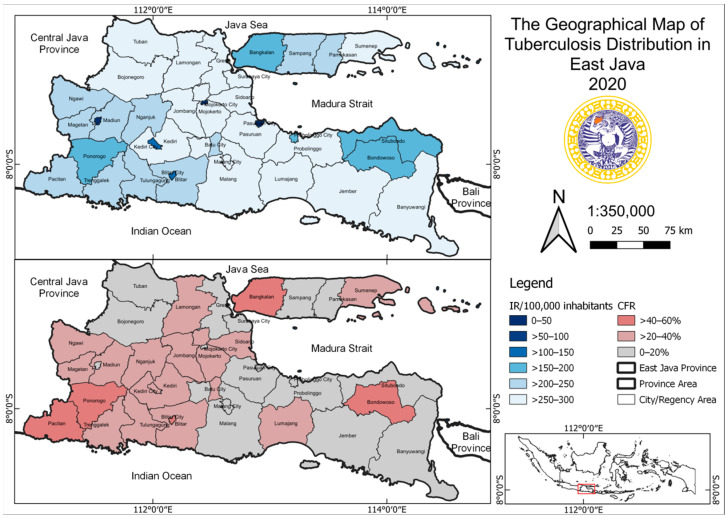
The geographical map of tuberculosis distribution in East Java Province in 2020.

**Figure 3:**
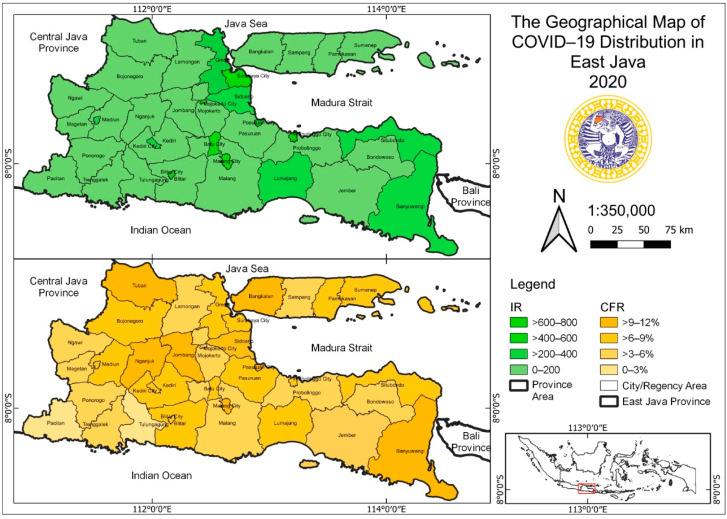
The geographical map of COVID-19 distribution in East Java Province in 2020.

The geographical maps of the incidence of tuberculosis and COVID-19 are shown in Figure 2 and Figure 3.

## 4. Discussion

This study showed no significant correlation between the incidence rate and case fatality rate for tuberculosis (*p* = 0.912; *p* < 0.05). The incidence rate of tuberculosis in East Java Province (586.24/100,000 inhabitants) in 2020 was higher than the national incidence rate (301/100,000 inhabitants). Furthermore, there was a decreasing incidence rate of tuberculosis in 2020 (95.49/100,000 inhabitants) compared to that in 2019 (95.925/100,000 inhabitants) [7]. The global incidence rate of tuberculosis in 2020 was 127/100,000 inhabitants [8]. This showed that the incidence rate in East Java needs to be properly managed. Meanwhile, one of the global milestones proposed by the World Health Organization (WHO) in 2020 is the reduction in incidence rate and tuberculosis deaths by 20% and 35%, respectively [8]. The overall case fatality rate of tuberculosis in East Java Province in 2020 was 3.6/100,000 inhabitants, while in 2019, it was 3.8/100,000 inhabitants [6].

The decrease in the incidence rate of tuberculosis was also in line with another report, where the global data showed a decline in three of the six WHO regions, namely Southeast Asia, the Eastern Mediterranean, and the Western Pacific. In 2020, Indonesia was one of the eight countries that reported a high TB rate, with an estimated incidence of, (1) India (26%), (2) China (8.5%), (3) Indonesia (8.4%), (4) the Philippines (6.0%), (5) Pakistan (5.8%), (6) Nigeria (4.6%), (7) Bangladesh (3.6%), and (8) South Africa (3.3%). The results showed that the incidence of tuberculosis was higher in men than in women. This is in line with the global data in 2020, which showed a tuberculosis rate of 56% in men, 33% in women, and 11% in children [8]. Moreover, these values were also obtained in the national tuberculosis survey in Vietnam [9].

The elimination of tuberculosis depends on the treatment success rate of infected people. In this study, some regencies in East Java with were discovered to have a TSR value below the target (<90%). A total of 20 regencies with a TSR of below 90% need to benefit from public health promotion and sensitize tuberculosis patients to complete their treatment. Indonesia can adopt one of the efforts used to increase the treatment success/completion rate in India through Private Provider Interface Agencies (PPIAs). In India, the effort was effective in increasing tuberculosis notification rates, testing, and treatment success rates [10,11]. PPIAs can provide interventions related to patient care, training physicians, tuberculosis diagnostics, treatment monitoring, and tuberculosis medicines [12].

Tuberculosis and COVID-19 are airborne diseases; the infection affects the lungs and has similar symptoms. Moreover, COVID-19 symptoms include fever or chills, cough, shortness of breath or difficulty breathing, fatigue and headache, muscle or body aches, loss of new taste or smell, sore throat, stuffy or runny nose, nausea, vomiting, and diarrhea. Meanwhile, tuberculosis symptoms are coughing up phlegm or blood, a cough that lasts more than 2 months, appetite and weight loss, chest pain, chills, fever or night sweats, and fatigue [13]. Previous studies have demonstrated an association between tuberculosis and COVID-19. Both active and a previous history of tuberculosis seem to be related to an increased risk for the development of COVID-19, and aggravate the prognosis of infection [11,14,15,16,17,18]. The damage caused by TB infection in the lungs exacerbates its impact on local immunity and increases the body’s susceptibility to airborne pathogens [19]. This increases the risk of COVID-19 developing in patients with a current or previous history of TB. Tuberculosis was found to be associated with a 2.10-fold increase in the risk of severe COVID-19 disease. In patients with previous respiratory disease, lung function can be impaired and a low resistance to viral infections can form, which can develop into acute respiratory distress syndrome (ARDS) [20,21].

In 2020, when all countries were affected by COVID-19, it was reported that there were 84,140 cases in East Java, Indonesia, where the number of infected females was higher than the number for males. This was not in line with the report from Peru, where the incidence rate in females was higher than in that males. COVID-19 can affect any age group due to its fast transmission rate; however, this study discovered that the highest incidence rate was in the age group between 46 and 59 years old. This was in line with the national survey in Peru, where the highest incidence was in people older than 50 years [22].

The elimination of tuberculosis has three pillars and components, namely: (1) integrated, patient-centered care, and prevention, (2) bold policies and supportive systems, (3) and intensified research and innovation. The first pillars can be translated into four activities: (a) early diagnosis of tuberculosis, including testing on universal drug susceptibility, screening of close contact groups, (b) treating and supporting people with tuberculosis to complete their treatment, (c) collaborative care that manages the comorbidities, (d) preventive treatment of people at high risk [8].

COVID-19 and tuberculosis data showed that there is a possibility of coinfection in a patient, where an individual can simultaneously be affected by both illnesses. However, the limitation of this study is its inability to capture co-infected patients or those who are only infected with one of the diseases. Therefore, clinicians should be concerned with chronic diseases in patients, such as coinfection with both COVID-19 and tuberculosis. This is because the coinfection with both diseases was already reported by studies in China [23,24], USA [25], and Italy [15,26], with various clinical characteristics.

Coinfection between tuberculosis and COVID-19 could have an important impact on the public health sector. In another case, public health also has an important role in combatting COVID-19 through various interventions, including physical distancing, self-quarantine, travel restrictions, a semi-lockdown, practicing good personal hygiene, eating nutritious food, increasing case-tracking, and vaccine development [27]. People with underlying respiratory diseases should be considered during the pandemic, to prevent its exacerbation [28].

Lung macrostructural changes caused by pulmonary tuberculosis affect the function and defence of the lower respiratory tract. This condition can be complicated due to the consequences of the inflammatory response exacerbated by SARS-CoV-2, such as oedema [18,29,30]. The reported complications of tuberculosis with COVID-19 coinfection that are hypoxemia, respiratory failure, acute respiratory distress syndrome (ARDS), the need for non-invasive ventilation, glucose abnormalities, and longer lengths of hospital stay, with a maximum of 130 days, and recurrent or concurrent bacterial infections [19,31,32,33,34]. The risk of recovery in COVID-19 patients with tuberculosis is 25% lower [16]. Meanwhile, the risk of mortality for COVID-19 patients with early treatment of pulmonary tuberculosis is 2.5 times higher, and a previous history of tuberculosis has a 50% higher mortality risk [17].

Previous studies showed that pulmonary tuberculosis patients had an increased susceptibility to COVID-19 infection and showed an increase in the severity of symptom development [14]. Cumulative research has reported 80 pulmonary tuberculosis patients with COVID-19 coinfection from China, India, Belgium, Brazil, France, Italy, Russia, Spain, Switzerland and Singapore. The highest case of tuberculosis sufferers presenting with COVID-19 coinfection occurred in Italy [35].

One of the principal efforts to suppress the transmission of tuberculosis in a community is the active case finding (ACF). Case finding could be deciphering this kind of activity, including house-to-house surveys, massive surveillance, increasing case-finding, and out-patient case detection, specifically in high-risk groups [36]. Historical results showed that mass radiography could screen 2000 cases in over 2 million individuals [37]. Sufficient case finding could lead to the successfully treatment of tuberculosis. In certain cases, case-finding could also find HIV-TB-coinfected patients, as mentioned in Nigeria, which could locate 109 HIV-TB infected patients. In Nigeria, successful treatment was proven to be associated with newly registered patients [38].

Health facilities were also a predictor of the treatment success rate for tuberculosis. According to this, intersectoral collaborations with the private sector should be increased. As in another report, private health facilities had a more successful treatment rate than public health facilities [39,40]. The other challenge in tuberculosis management was drug resistance; interestingly, if the treatment success rate reaches 85%, this could lead to a reduction in transmission, drug resistance, TB prevalence, and TB incidence. In East Java Province, the number of health facilities, specifically primary health centers, is 968 units. However, not all primary health centers have the same capacity to tackle tuberculosis, especially during the pandemic, when the all the health facilities were focused on COVID-19. In addition, Indonesia also had an endemic for another tropical disease, which was neglected [41].

Drug resistance in tuberculosis become a challenge in TB management, due to the increasing number of mechanisms that were involved. This phenomenon led to difficultes in the development of diagnostic procedures. However, whole-genome sequencing (WGS) may can help to identify polymorphisms related to drug resistance. In another case, the capacity of laboratories which can conduct WGS in developing countries is still limited [42]. During the pandemic, whole-genome sequencing (WGS) was introduced, specifically to understand the new variant of COVID-19, which continuously increased. According to the latest information, the COVID-19 variants can be classified into three groups, namely, variants of concern (VOC), variants of interest (VOI) and high-consequence variants (VOHC). This classification was formulated according to the capacity and the impact on global public health [41].

## 5. Conclusions

In conclusion, these respiratory diseases still need to be evaluated, specifically in a clinical setting. Although there have been major advances in infectious disease control in recent years, the number of infectious diseases, including TB and COVID-19 in East Java Province, is still a concern. Therefore, a retrospective study was carried out to evaluate the recent epidemiology of the infectious disease in East Java, which is the second largest province in Indonesia and consisted of 38 regencies with large populations. The results showed that the incidence rate of tuberculosis was higher in males than females, while the rate of COVID-19 was higher in females than in males. The highest cases of COVID-19 were discovered in the age group between 46 and 59 years old. The highest COVID-19 incidence rate was found in Mojokerto City, while the lowest was found in Sampang. Tuberculosis and COVID-19 are the pulmonary diseases which need to be considered. According to this study, the treatment success rate of tuberculosis needs to be improved to achieve a decline in case fatality rates and transmission in the community. During the pandemic, it is important to increase active tuberculosis case detection in order to minimize community transmission.

### Limitations

The limitation of this study was the limited data that could be provided, due to the data source, which could only capture regional surveillance data.

## Figures and Tables

**Table 1:** Distribution of incidence rate (IR) and case fatality rate of TB and COVID-19 in 2020.

No.	Regency	TB Infection		COVID-19 Infection	
		IR *	CFR	IR *	CFR
1.	Pacitan	51.8	1.9%	136.5	2.8%
2.	Ponorogo	103.69	1.3%	142.2	4.0%
3.	Trenggalek	57.63	0.9%	148.9	4.4%
4.	Tulungagung	78.70	1.4%	129.8	**2.1%**
5.	Blitar	55.08	1.7%	156.5	8.0%
6.	Kediri	0.1	0.8%	152.8	7.8%
7.	Malang	**0.07**	1.3%	56.5	5.7%
8.	Lumajang	0.11	0.8%	230.9	6.6%
9.	Jember	0.12	0.7%	180.4	4.7%
10.	Banyuwangi	0.12	2.0%	253.4	9.3%
11.	Bondowoso	114.54	1.0%	193.1	5.3%
12.	Situbondo	142.47	0.7%	248.5	7.0%
13.	Probolinggo	0.1	3.2%	188.4	5.1%
14.	Pasuruan	0.11	0.8%	133.8	7.2%
15.	Sidoarjo	0.11	1.2%	349.7	6.6%
16.	Mojokerto	0.1	0.6%	132.0	3.0%
17.	Jombang	0.1	1.4%	182.8	10.6%
18.	Nganjuk	68.02	1.0%	103.8	9.9%
19.	Madiun	86.28	1.8%	57.0	7.4%
20.	Magetan	75.04	1.1%	150.1	5.0%
21.	Ngawi	90.95	1.4%	71.1	5.9%
22.	Bojonegoro	0.11	1.4%	102.8	7.2%
23.	Tuban	0.11	1.7%	152.1	10.6%
24.	Lamongan	0.13	1.0%	146.0	6.0%
25.	Gresik	0.11	1.3%	313.9	6.6%
26.	Bangkalan	100.48	0.7%	100.6	9.4%
27.	Sampang	82.71	1.2%	**52.5**	5.2%
28.	Pamekasan	82.07	1.2%	86.7	8.1%
29.	Sumenep	0.15	2.1%	110.3	6.1%
30.	Kediri City	199.92	1.2%	248.3	7.2%
31.	Blitar City	166.67	1.1%	467.1	5.4%
32.	Malang City	0.16	0.6%	422.8	9.9%
33.	Probolinggo city	145.59	**7.0%**	611.7	7.3%
34.	Pasuruan City	258.45	1.1%	497.1	**11.0%**
35.	Mojokerto City	254.06	0.9%	**789.9**	5.7%
36.	Madiun City	**296.51**	1.6%	227.7	7.7%
37.	Surabaya City	0.14	**0.4%**	625.3	6.9%
38.	Batu City	88.46	0.5%	498.7	8.3%
	Average	68.44	0.013	232.93	0.067

* IR: Incidence rate per 100,000 populations. CFR: Case Fatality Rate. Bold font indicated the highest or the lowest data.

**Table 2:** Case recovery rate (CRR) of TB and COVID-19 in East Java Province in 2020.

No.	Regency	Case Recovery Rate (CRR)	
		TB (%)	COVID-19 (%)
1.	Pacitan	81.5	79.71
2.	Ponorogo	84.5	81.93
3.	Trenggalek	91.2	83.92
4.	Tulungagung	64.4	71.86
5.	Blitar	75.9	85.61
6.	Kediri	92.2	80.49
7.	Malang	78.2	92.03
8.	Lumajang	52.4	83.58
9.	Jember	86	80.96
10.	Banyuwangi	76.2	83.24
11.	Bondowoso	68.7	90.82
12.	Situbondo	85.4	87.61
13.	Probolinggo	83.5	85.08
14.	Pasuruan	57.8	83.84
15.	Sidoarjo	74.3	**92.62**
16.	Mojokerto	78	89.24
17.	Jombang	81.1	85.25
18.	Nganjuk	74.1	81.58
19.	Madiun	73.4	65.89
20.	Magetan	**96.6**	84.74
21.	Ngawi	84.9	78.81
22.	Bojonegoro	93.1	75.13
23.	Tuban	75	**64.8**
24.	Lamongan	82.9	86.52
25.	Gresik	76.7	91.59
26.	Bangkalan	92.2	74.9
27.	Sampang	58.3	83.84
28.	Pamekasan	84.5	68.44
29.	Sumenep	77.4	74.19
30.	Kediri City	74.8	79.53
31.	Blitar City	84.3	86.2
32.	Malang City	63.2	81.42
33.	Probolinggo city	59.6	73.59
34.	Pasuruan City	84.6	84.23
35.	Mojokerto City	58.7	92.59
36.	Madiun City	91.4	75.74
37.	Surabaya City	72.5	92.32
38.	Batu City	**11.3**	88.68
	Average	75.81	82.17

Source: East Java Health Profile 2020. Bold font indicated the highest or the lowest data.

**Table 3:** Distribution of TB and COVID-19 cases according to sex.

No.	Regency	TB (Number of People) *		COVID-19 (Number of People) *	
		Male	Female	Male	Female
1.	Pacitan	170	118	386	373
2.	Ponorogo	530	374	674	566
3.	Trenggalek	237	165	491	548
4.	Tulungagung	463	358	636	718
5.	Blitar	366	275	913	908
6.	Kediri	815	727	1066	1349
7.	Malang	1026	802	761	720
8.	Lumajang	630	499	1168	1244
9.	Jember	1640	**1407**	2076	2369
10.	Banyuwangi	1119	886	1902	2197
11.	Bondowoso	477	415	503	1001
12.	Situbondo	531	446	738	966
13.	Probolinggo	561	604	926	1287
14.	Pasuruan	910	860	1130	1061
15.	Sidoarjo	1440	1080	4015	3965
16.	Mojokerto	615	465	702	785
17.	Jombang	706	582	1057	1262
18.	Nganjuk	417	302	500	597
19.	Madiun	345	245	**198**	**192**
20.	Magetan	271	201	456	488
21.	Ngawi	441	314	291	299
22.	Bojonegoro	820	611	599	688
23.	Tuban	714	572	844	946
24.	Lamongan	858	637	834	903
25.	Gresik	839	624	1878	2285
26.	Bangkalan	534	465	507	493
27.	Sampang	467	351	234	285
28.	Pamekasan	409	320	350	420
29.	Sumenep	952	660	480	725
30.	Kediri City	319	259	290	428
31.	Blitar City	134	104	367	300
32.	Malang City	739	638	1817	1882
33.	Probolinggo city	177	171	768	694
34.	Pasuruan City	289	232	502	500
35.	Mojokerto City	189	141	489	537
36.	Madiun City	327	199	203	201
37.	Surabaya City	**2305**	1846	**8889**	**9275**
38.	Batu City	**93**	**92**	545	498
	Total	23,875	19,047	40,185	43,955

* Source: East Java Health Profile 2020. Bold font indicated the highest or the lowest data.

**Table 4:** The incidence rate of TB and COVID-19 according to sex group.

No.	Regency	Incidence Rate of TB		Incidence Rate of COVID-19	
		Male	Female	Male	Female
1.	Pacitan	**57.88**	**40.36**	131.42	127.57
2.	Ponorogo	111.75	78.73	142.12	119.14
3.	Trenggalek	64.51	45.36	133.65	150.65
4.	Tulungagung	84.91	65.75	116.64	131.86
5.	Blitar	59.37	45.29	148.09	149.53
6.	Kediri	98.68	89.82	129.08	166.66
7.	Malang	76.69	60.91	56.88	54.68
8.	Lumajang	113.81	88.21	210.99	219.91
9.	Jember	129.65	110.63	164.11	186.28
10.	Banyuwangi	130.84	103.88	222.40	257.59
11.	Bondowoso	124.80	105.35	131.60	254.11
12.	Situbondo	157.68	127.72	219.15	276.62
13.	Probolinggo	98.65	103.45	162.83	220.43
14.	Pasuruan	113.22	107.20	140.59	132.25
15.	Sidoarjo	137.33	104.43	382.90	383.38
16.	Mojokerto	109.25	83.59	124.70	141.12
17.	Jombang	106.23	89.06	159.04	193.13
18.	Nganjuk	75.10	55.05	90.04	108.82
19.	Madiun	93.52	65.26	53.67	**51.14**
20.	Magetan	82.12	58.98	138.17	143.20
21.	Ngawi	102.09	71.68	67.37	68.25
22.	Bojonegoro	125.44	94.30	91.63	106.18
23.	Tuban	119.33	95.39	141.06	157.75
24.	Lamongan	127.55	94.87	123.98	134.48
25.	Gresik	127.07	95.86	284.42	351.04
26.	Bangkalan	102.15	86.50	96.98	91.70
27.	Sampang	96.95	71.92	**48.58**	58.40
28.	Pamekasan	98.09	73.89	83.94	96.98
29.	Sumenep	175.41	113.46	88.44	124.63
30.	Kediri City	222.12	180.89	201.93	298.92
31.	Blitar City	180.55	138.79	494.50	400.36
32.	Malang City	175.99	150.50	432.72	443.96
33.	Probolinggo city	148.95	141.53	646.31	574.41
34.	Pasuruan City	277.76	**223.16**	482.48	480.95
35.	Mojokerto City	287.74	211.24	**744.46**	**804.51**
36.	Madiun City	**342.62**	199.53	212.69	201.54
37.	Surabaya City	161.74	127.39	623.72	640.03
38.	Batu City	86.67	87.00	507.92	470.94
	Average	131.16	102.29	221.87	236.13

Bold font indicated the highest or the lowest data.

**Table 5:** The treatment success rate of TB in 2020.

No.	Regency	Treatment Success Rate Tuberculosis (%)
1.	Pacitan	82.96%
2.	Ponorogo	85.49%
3.	Trenggalek	94.03%
4.	Tulungagung	89.20%
5.	Blitar	88.85%
6.	Kediri	79.65%
7.	Malang	86.06%
8.	Lumajang	92.27%
9.	Jember	87.55%
10.	Banyuwangi	90.17%
11.	Bondowoso	**65.89%**
12.	Situbondo	93.28%
13.	Probolinggo	91.41%
14.	Pasuruan	89.34%
15.	Sidoarjo	91.08%
16.	Mojokerto	89.40%
17.	Jombang	88.02%
18.	Nganjuk	79.17%
19.	Madiun	89.82%
20.	Magetan	**95.97%**
21.	Ngawi	92.54%
22.	Bojonegoro	93.57%
23.	Tuban	92.33%
24.	Lamongan	94.89%
25.	Gresik	91.69%
26.	Bangkalan	92.26%
27.	Sampang	85.67%
28.	Pamekasan	90.58%
29.	Sumenep	89.44%
30.	Kediri City	95.46%
31.	Blitar City	81.07%
32.	Malang City	85.14%
33.	Probolinggo city	72.69%
34.	Pasuruan City	91.51%
35.	Mojokerto City	81.05%
36.	Madiun City	93.66%
37.	Surabaya City	90.61%
38.	Batu City	71.78%
	Average	87.78%

Bold font indicated the highest or the lowest data.

**Table 6:** The Results of Spearman Correlation and Mann–Whitney test.

**Spearman Correlation Test (*p*-Value Was Calculated as Significant If *p* < 0.05)**			
***p*-Value**	**CFR_TB**	**CFR_COVID-19**	**TSR_TB**
IR_TB	0.912		0.795
IR_COVID-19		0.219	
CRR_TB	0.698		
CRR_COVID-19		0.164	
IR_TB			0.795
CFR_TB			0.659
**Mann–Whitney Test**			
***p*-Value**	**IR_TB_Female**	**IR_COVID-19_Female**	
IR_TB_Male	0.202		
IR_COVID-19 Male		0.942	

**Table 7:** The results of Pearson correlation test of demographical factors related to the incidence rate of TB.

Bivariate Analysis of Geographical Factors with the Incidence Rate of Tuberculosis			Correlation between Two Variables
*Variables*	*p*-Value	*r*-Correlation	
Ratio of public health centre	0.001 *	0.53	Strong positive correlation
Ratio of doctor	0.000 *	0.65	Strong positive correlation
Ratio of nurse	0.000 *	0.70	Strong positive correlation
Ratio of midwifery	0.000 *	0.74	Strong positive correlation
Ratio of public health workers	0.002 *	0.49	Enough positive correlation
Ratio of environmental health workers	0.000 *	0.71	Strong positive correlation
Ratio of nutrinionist	0.000 *	0.72	Strong positive correlation
Ratio of pharmacist	0.000 *	0.67	Strong positive correlation
Incidence rate of HIV	0.002 *	0.48	Enough positive correlation

* A *p*-value less than 0.05 is statistically significant.

**Table 8:** The results of Mann–Whitney test for the comparison between case recovery rate of tuberculosis and case recovery rate of COVID-19.

*p*-Value of Mann–Whitney Test between Case Recovery Rate of TB and Case Recovery Rate of COVID-19			
	*p*-Value		
CRR TB	0.051		

**Table 9:** The results of Pearson Correlation test of demographical factors related to the incidence rate of COVID-19.

Bivariate Analysis of Geographical Factors with the Incidence Rate of COVID-19			Correlation between Two Variables
*Variables*	*p*-Value	*r*-Correlation	
Ratio of public health centre	0.44	0.13	Very low positive correlation
Ratio of doctor	0.001 *	0.7	Strong positive correlation
Ratio of nurse	0.001 *	0.64	Strong positive correlation
Ratio of midwifery	0.27	0.18	Very low positive correlation
Ratio of public health workers	0.01 *	0.42	Enough positive correlation
Ratio of environmental health workers	0.01 *	0.41	Enough positive correlation
Ratio of nutrinionist	0.001 *	0.56	Strong positive correlation
Ratio of pharmacist	0.001 *	0.55	Strong positive correlation
Incidence rate of HIV	0.002 *	0.49	Enough positive correlation

* A *p*-value less than 0.05 is statistically significant.

## Data Availability

The datasets used and/or analysed during the present study are available from the corresponding author on reasonable request.

## Supplementary Materials

The following supporting information can be downloaded at: https://www.mdpi.com/article/10.3390/tropicalmed7060083/s1, Table S1. The demographical factor analysis (Ratio number of public health center, ratio number of doctor, and ratio number of nurse/100,000 populations); Table S2. The demographical factor analysis (Ratio number of midwifery, ratio number of public health workers, and ratio number of environmental health workers/100,000 populations); Table S3. The demographical factor analysis (Ratio number of nutritionist, ratio number of pharmacist, and incidence rate of HIV/100,000 populations).
