# Supplementary material for: Comparison Epidemiology between Tuberculosis and COVID-19 in East Java Province, Indonesia: An Analysis of Regional Surveillance Data in 2020
Source: Trop Med Infect Dis. 2022 May 27;7(6):83. doi: 10.3390/tropicalmed7060083 (PMC9230370; doi:10.3390/tropicalmed7060083)
Supplement: Supplementary file 1 [file tropicalmed-07-00083-s001.zip › tropicalmed-1703767-supplementary.pdf]

Tropical Medicine and Infectious Disease

MDPI

Supplementary Materials: Comparison Epidemiology between Tuberculosis and COVID-19 in East Java Province, Indonesia: An Analysis of Regional Surveillance Data in 2020

Budi Utomo [1,*], Chow Khuen Chan [2], Ni Made Mertaniasih [3,4], Soedarsono Soedarsono [5], Shifa Fauziyah [6], Teguh Hari Sucipto [7], Febriana Aquaresta [8], Dwinka Syafira Eljatin [9] and I Made Dwi Mertha Adnyana [9]

Table S1. The demographical factor analysis (Ratio number of public health center, ratio number of doctor, and ratio number of nurse/100,000 populations)

No.	Residence	Ratio Number of Public Health Centre/100,000 Inhabitants	Ratio Number of Doctor/100,000 Inhabitants	Ratio Number of Nurse
1	Pacitan	4.32	4.68	113.13
2	Ponorogo	3.56	5.39	157.14
3	Trenggalek	3.15	3.87	128.15
4	Tulungagung	3.07	4.60	140.24
5	Blitar	2.06	3.69	72.87
6	Kediri	2.34	5.95	83.22
7	Malang	1.49	3.93	104.39
8	Lumajang	2.39	4.69	128.55
9	Jember	2.03	5.57	93.70
10	Banyuwangi	2.78	6.61	108.73
11	Bondowoso	3.21	6.16	137.52
12	Situbondo	2.92	6.12	132.40
13	Probolinggo	2.81	4.00	85.03
14	Pasuruan	2.02	3.54	74.31
15	Sidoarjo	1.14	8.63	132.68
16	Mojokerto	2.40	4.62	121.54
17	Jombang	2.68	5.36	137.48
18	Nganjuk	1.89	3.22	112.96
19	Madiun	3.80	5.26	113.49
20	Magetan	3.50	5.09	109.06
21	Ngawi	2.89	7.47	139.25
22	Bojonegoro	2.88	4.71	107.67
23	Tuban	2.80	4.42	78.42
24	Lamongan	2.77	4.37	156.64
25	Gresik	2.41	6.86	128.16
26	Bangkalan	2.21	3.02	101.29
27	Sampang	2.12	2.02	47.32
28	Pamekasan	2.25	2.81	128.91
29	Sumenep	2.75	2.93	101.80
30	Kediri City	3.11	38.74	493.59
31	Blitar City	2.10	27.31	478.30
32	Malang City	1.83	17.72	379.02
33	Probolinggo city	2.51	20.08	235.12
34	Pasuruan City	3.97	19.84	236.13
35	Mojokerto City	4.62	26.18	597.42
36	Madiun City	3.38	18.60	620.63
37	Surabaya City	2.17	15.04	303.43
38	Batu City	2.39	12.91	217.10

Table S2. The demographical factor analysis (Ratio number of midwifery, ratio number of public health workers, and ratio number of environmental health workers/100,000 populations).

No.	Residence	Ratio Number of Midwifery	Ratio Number of Public Health Workers	Ratio Number of Environmental Health Workers
1	Pacitan	67.27	8.63	9.71
2	Ponorogo	69.85	5.39	5.28
3	Trenggalek	57.05	6.88	7.31
4	Tulungagung	58.38	4.12	4.31
5	Blitar	48.20	2.49	2.41
6	Kediri	52.40	3.73	4.81
7	Malang	32.90	0.95	2.14
8	Lumajang	56.47	6.70	3.64
9	Jember	0.04	4.96	1.14
10	Banyuwangi	0.07	2.60	2.29
11	Bondowoso	79.74	3.72	4.37
12	Situbondo	97.41	5.54	4.08
13	Probolinggo	61.03	2.81	2.55
14	Pasuruan	45.80	3.36	2.63
15	Sidoarjo	43.25	5.26	2.19
16	Mojokerto	58.06	2.57	3.20
17	Jombang	90.66	5.68	3.55
18	Nganjuk	73.23	1.99	3.78
19	Madiun	62.15	7.02	5.12
20	Magetan	84.26	4.61	6.20
21	Ngawi	76.73	6.38	5.90
22	Bojonegoro	79.63	4.55	3.67
23	Tuban	41.72	1.53	3.23
24	Lamongan	81.56	1.43	1.60
25	Gresik	67.85	4.30	2.26
26	Bangkalan	99.88	1.11	1.61
27	Sampang	48.23	3.94	1.92
28	Pamekasan	74.87	5.18	3.26
29	Sumenep	88.70	4.03	1.92
30	Kediri City	153.92	10.72	9.68
31	Blitar City	109.25	8.40	9.10
32	Malang City	88.93	7.54	6.97
33	Probolinggo city	81.58	5.86	8.79
34	Pasuruan City	111.12	3.47	6.94
35	Mojokerto City	133.96	9.24	10.01
36	Madiun City	156.15	24.80	18.04
37	Surabaya City	0.05	14.11	7.71
38	Batu City	53.08	17.69	2.87

Table S3. The demographical factor analysis (Ratio number of nutritionist, ratio number of pharmacist, and incidence rate of HIV/100,000 populations).

No.	Residence	Ratio Number of Nutritionist	Ratio Number of Pharmacist	IR HIV
1	Pacitan	12.41	10.43	2.88
2	Ponorogo	6.65	7.11	14.45
3	Trenggalek	10.46	6.16	4.59
4	Tulungagung	7.57	9.59	22.81
5	Blitar	6.27	12.46	7.99
6	Kediri	7.21	13.99	10.95

7	Malang	4.50	4.27	10.11
8	Lumajang	8.14	8.33	79.16
9	Jember	4.31	2.97	28.86
10	Banyuwangi	6.12	10.94	31.21
11	Bondowoso	8.22	3.34	16.95
12	Situbondo	8.90	5.54	35.73
13	Probolinggo	4.85	3.92	13.11
14	Pasuruan	4.27	3.24	16.24
15	Sidoarjo	5.13	9.03	17.79
16	Mojokerto	4.79	5.33	5.68
17	Jombang	7.02	6.07	14.11
18	Nganjuk	5.01	8.04	15.42
19	Madiun	7.17	7.17	15.21
20	Magetan	6.04	5.56	11.45
21	Ngawi	7.59	6.02	11.68
22	Bojonegoro	5.35	9.58	13.34
23	Tuban	4.76	3.74	2.89
24	Lamongan	5.63	5.72	12.53
25	Gresik	4.07	7.31	6.03
26	Bangkalan	3.22	3.32	2.21
27	Sampang	4.04	0.81	9.40
28	Pamekasan	5.18	7.32	4.17
29	Sumenep	4.39	1.56	1.28
30	Kediri City	20.06	26.98	80.94
31	Blitar City	20.31	47.62	83.33
32	Malang City	19.20	23.89	48.81
33	Probolinggo city	12.55	17.57	47.69
34	Pasuruan City	11.91	19.35	25.30
35	Mojokerto City	23.87	46.19	50.81
36	Madiun City	32.13	72.72	65.39
37	Surabaya City	10.67	17.08	27.06
38	Batu City	12.91	25.34	9.09
